# Imagine, Discover, Inspire: Proceedings of the 4th International Conference of the Trisomy 21 Research Society

**DOI:** 10.1007/s12017-024-08824-y

**Published:** 2025-01-05

**Authors:** Lisi Flores-Aguilar, Eric D. Hamlett, Paula Araya, Eugenio Barone, Anita Bhattacharyya, Maria Carmona-Iragui, Li Chan, Brad Christian, Alberto C. S. Costa, Floriana Costanzo, Laura Del Hoyo Soriano, Mara Dierssen, Evan E. Eichler, Elizabeth Fisher, Matthew Galbraith, Sujay Ghosh, Sandra Gimenez, Faycal Guedj, Sandra Guidi, Maria Florencia Iulita, William Mobley, Maria Chiara Pelleri, Marie-Claude Potier, Karen R. Rabin, Angela Rachubinski, Anne-Sophie Rebillat, Eric Rubenstein, Hannah Saternos, Lorena Sordo, Andre Strydom, Natalia Valle-Tamayo, Katherine A. Waugh, Eugene Yu, Ella Zeldich, Jorge Busciglio, Elizabeth Head

**Affiliations:** 1 Department of Pathology and Laboratory Medicine, University of California, Irvine, Irvine, CA USA; 2 Department of Pathology and Laboratory Medicine, Medical University of South Carolina, Charleston, SC USA; 3 Department of Pediatrics, Section of Developmental Pediatrics, Linda Crnic Institute for Down Syndrome, University of Colorado Anschutz Medical Campus, Aurora, CO USA; 4 Department of Biochemical Sciences “A. Rossi-Fanelli, Sapienza University of Rome, Rome, Italy; 5 Waisman Center, University of Wisconsin-Madison, Madison, WI USA; 6 Department of Cell and Regenerative Biology, School of Medicine and Public Health, University of Wisconsin-Madison, Madison, WI USA; 7 Memory Unit, Department of Neurology, Hospital de la Santa Creu i Sant Pau, Biomedical Research Institute Sant Pau, Universitat Autònoma de Barcelona, Barcelona, Spain; 8 Barcelona Down Medical Center, Fundació Catalana Síndrome de Down, Barcelona, Spain; 9 Centre for Endocrinology, William Harvey Research Institute, Queen Mary University of London, London, UK; 10 Department of Medical Physics and Psychiatry, University of Wisconsin-Madison, Madison, WI USA; 11 Department of Psychiatry, Case Western Reserve University, Cleveland, OH USA; 12 Child and Adolescent Neuropsychiatry Unit, Bambino Gesù Children’s Hospital, IRCCS, Rome, Italy; 13 MIND Institute and Department of Psychiatry and Behavioral Sciences, University of California Davis Health, 2828 50Th St., Sacramento, CA USA; 14 Centre for Genomic Regulation (CRG), The Barcelona Institute of Science and Technology, Barcelona, Spain; 15 Human Pharmacology and Clinical Neurosciences Research Group, Neurosciences Research Program, Hospital del Mar Medical Research Institute (IMIM), Barcelona, Spain; 16 Department of Genome Sciences, School of Medicine, University of Washington, Seattle, WA USA; 17 Howard Hughes Medical Institute, University of Washington, Seattle, WA USA; 18 Department of Neuromuscular Diseases, LonDownS: London Down Syndrome Consortium, Queen Square Institute of Neurology, University College London, London, UK; 19 Department of Pharmacology, University of Colorado School of Medicine, Anschutz Medical Campus, Aurora, CO USA; 20 Cytogenetics and Genomics and Down Syndrome Research Unit, Department of Zoology, University of Calcutta, Kolkata, India; 21 Multidisciplinary Sleep Unit, Respiratory Department, Hospital de la Santa Creu i Sant Pau, Barcelona, Spain; 22 Section On Prenatal Genomics and Fetal Therapy, Center for Precision Health Research, National Human Genome Research Institute, National Institutes of Health, Bethesda, MD USA; 23 Department of Neurosciences, University of California, San Diego, La Jolla, CA USA; 24 Department of Biomedical and Neuromotor Sciences, Physiology Building, University of Bologna, Bologna, Italy; 25 Institut du Cerveau-Paris Brain Institute-ICM, Institut National de la Santé et de la Recherche Médicale, Centre National de la Recherche Scientifique, Assistance Publique-Hôpitaux de Paris, Hôpital de la Pitié Salpêtrière, Sorbonne Université, Paris, France; 26 Division of Pediatric Hematology-Oncology, Texas Children’s Cancer Center, Baylor College of Medicine, Houston, TX USA; 27 Institut Jérôme Lejeune, Paris, France; 28 Department of Epidemiology, Boston University School of Public Health, Boston, MA USA; 29 Department of Neurosurgery, School of Medicine, University of Colorado Anschutz Medical Campus, Aurora, CO USA; 30 Department of Forensic and Neurodevelopmental Sciences, Institute of Psychiatry, Psychology and Neuroscience, King’s College London, London, UK; 31 Center of Biomedical Investigation Network for Neurodegenerative Diseases, Madrid, Spain; 32 The Children’s Guild Foundation Down Syndrome Research Program, Department of Cancer Genetics and Genomics, Roswell Park Comprehensive Cancer Center, Buffalo, NY USA; 33 Department of Anatomy and Neurobiology, Boston University, Boston, MA USA; 34 Neurobiology and Behavior School of Biological Sciences, University of California, Irvine, CA USA

**Keywords:** Down syndrome, Preclinical research, Clinical research, Biomarkers, Intellectual disability, Conference proceedings

## Abstract

Down syndrome (DS) or trisomy 21 (T21) is present in a significant number of children and adults around the world and is associated with cognitive and medical challenges. Through research, the T21 Research Society (T21RS), established in 2014, unites a worldwide community dedicated to understanding the impact of T21 on biological systems and improving the quality of life of people with DS across the lifespan. T21RS hosts an international conference every two years to support collaboration, dissemination, and information sharing for this goal. In 2022, T21RS hosted an international conference in Long Beach, California, from June 9 to 12. The conference, attended by 483 people including scientists, families, self-advocates, and industry representatives from 17 countries, was a dynamic and interactive meeting that shared discoveries from international research teams. This summary highlights the scientific discoveries shared at the 4th T21RS meeting with the Imagine, Discover, Inspire theme.

## Introduction

An important mission of T21RS is to provide a distinguished forum for communicating and advancing preclinical and clinical research on DS. It seeks to ensure that advances in understanding the biology of DS are translated into interventions that promote health and well-being. The meeting in Long Beach provided an opportunity to showcase the Society’s vital work and to support person-to-person interactions for sharing ideas and planning new research activities. The T21RS international conference in Long Beach, California, was an exciting forum for researchers, families, self-advocates, and industry to come together and share information to benefit the quality of life for people with DS. The meeting focused on research across the lifespan of people with DS, from early life challenges to adulthood and aging. The meeting included research representing multidisciplinary approaches from genetics, molecular biology, in vitro studies of cells, and mouse models to human studies (clinical, cognitive, and biomarkers).

**Fig. 1:**
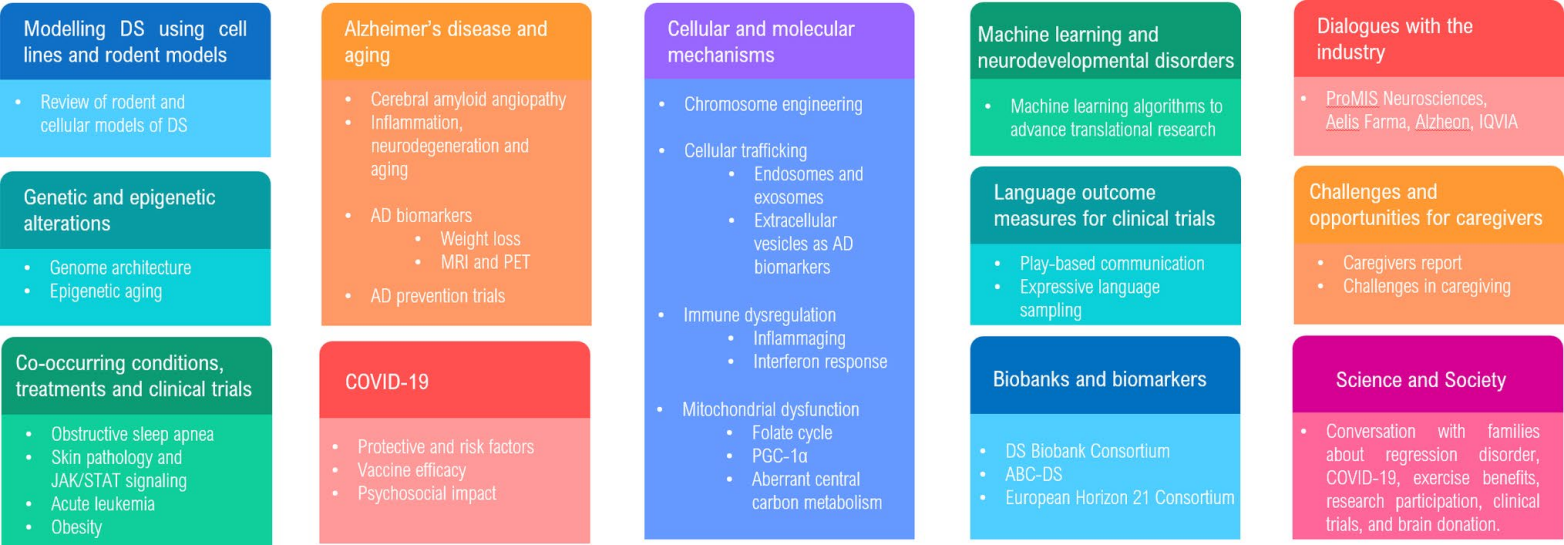
Topics covered at the T21RS Meeting

**Fig. 2:**
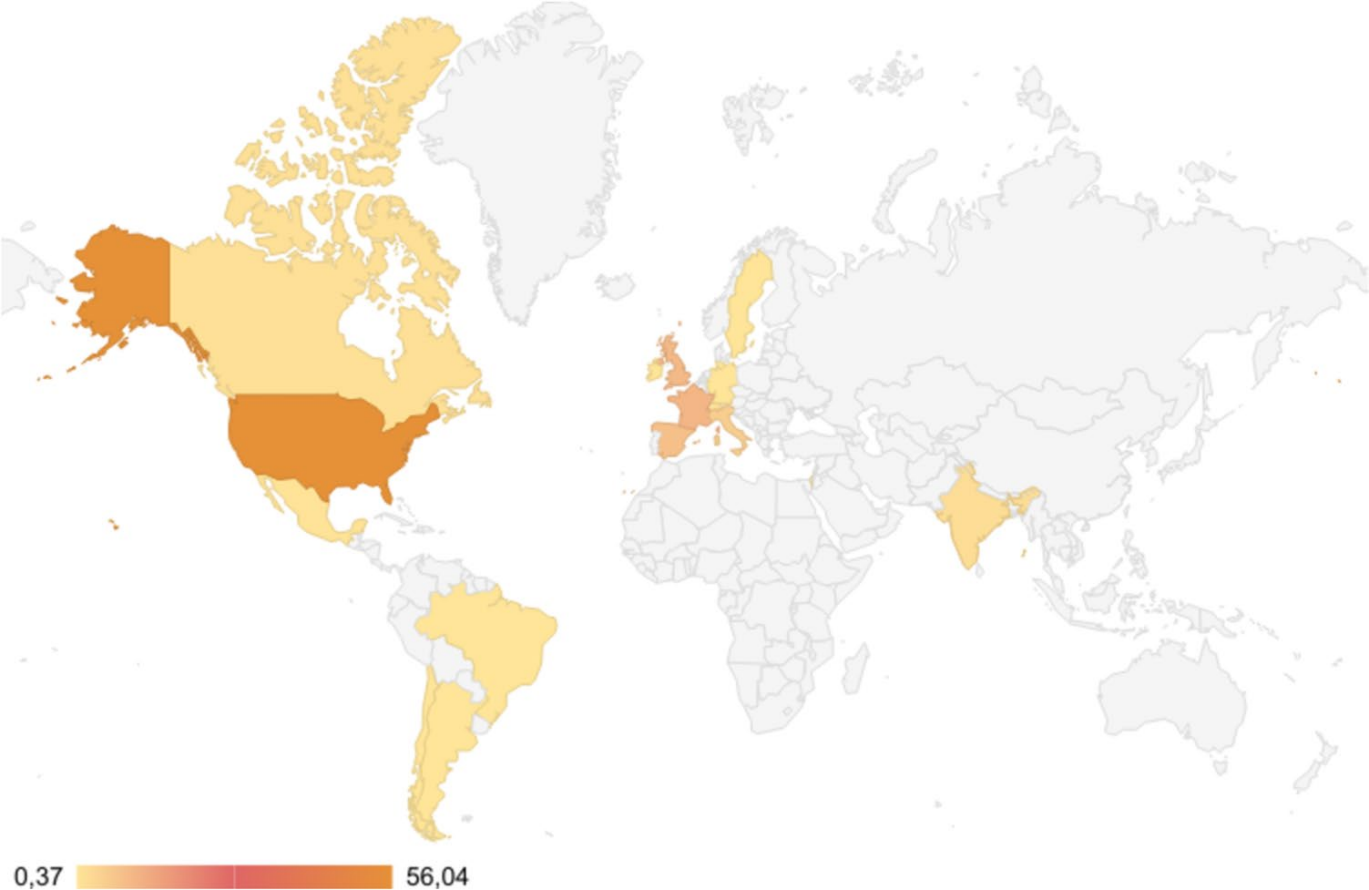
Countries represented by attendees at the T21RS Meeting

The meeting included several themes: Genomic and Epigenetic Mechanisms, Molecular and Cellular Mechanisms, Experimental Models, Cognition and Behavior, Neurodegeneration and Ageing, Neurodevelopment, Co-occurring Illnesses, Therapeutic intervention and Diagnosis and Evaluation (Fig. 1). We hosted four exciting plenary lectures, four satellite meetings, 20 symposia, nine nano symposia, and two dedicated sessions for junior investigators. We were delighted to receive 141 abstracts. We had exceptional international attendance from 483 people across 17 countries (Fig. 2), with 204 presenters (oral or poster presentations).

## Understanding and Using Models to Learn About DS

Scientists continue to use various models of DS to study the genetic and biological causes of DS-related medical conditions and to test potential therapies that could benefit individuals with DS. Recent innovations led to the development of new animal and cellular models along with analysis methods. The T21RS Preclinical Committee organized an informative satellite meeting to allow all those working with vertebrate models of DS to consider what they want from each model, how to use each model, its pros and cons, new models that have become available, and how to access the valuable list of models for T21RS members that can be found on the Preclinical Committee T21RS website pages.

Roger Reeves (USA) presented a brief history of mouse DS models and discussed new humanized and rat models of DS. Yann Herault (France) presented new rat and mouse DS models. Both presenters pointed out that studies of rats may offer distinct advantages, especially for behavioral testing, when compared to prey animals such as mice. In the final talk, Randall Roper (USA) described his findings regarding sexually dimorphic bone phenotypes in DS mouse models. Roper stressed that animal experiments must be reported per the latest ARRIVE (Animal Research: Reporting of In Vivo Experiments) guidelines to fully capture all relevant data. A short discussion followed the presentations and focused on in vivo models, including their advantages and limitations.

The next session focused on iPSC/cell, organoid, and tissue models. Hiruy Meharena (USA) presented the strengths and weaknesses of cell models and best practices to ensure rigor and reproducibility. Jean Delabar (France), Joe Lee (USA), and Marie-Claude Potier (France) illustrated the power of using cell lines in different types of studies, genetic and functional, to help understand mechanisms in DS and other disorders.

The next session tackled probably the most challenging areas to model—behavior and cognition. Mara Dierssen (Spain) organized this session and gave an excellent overview of the need for standardization, the difficulty of achieving this, and how the field might devise and validate reliable and sensitive behavioral and other tests to cross the species boundaries. Javier Zorrilla de San Martin (France) discussed the prefrontal cortex and its electrical readout during rodent cognition, Álvaro Fernández (Spain) talked about new techniques for cognitive interrogation using opto- and chemo-genetics and Tomer Illouz (Israel) presented a new computational approach to quantify rodent strategies in behavioral tasks.

Conclusions: The satellite was a stimulating and informative session that led to many new questions about modeling DS using rodent and cell models. Still, it showed that important new models and analysis methods are becoming available to help us further understand the many complex aspects of DS.

## Genomic and Epigenetic Studies in DS

### Genomic Alterations in DS

Researchers are exploring new technologies to study human genome regulation to gain novel insights into how DNA and RNA changes alter physiological and cellular functions for individuals with DS. Maria del Mar Dierssen Sotos (Spain) gave the Jerome Lejeune Presidential Plenary Lecture and proposed that genome-wide changes in gene expression outside of the trisomy may significantly contribute to DS phenotypes and thus merit further investigation. Her research suggests that the genomic impact beyond human chromosome 21 (Hsa21) is due to the reconfiguration of genome architecture driven by the presence of the extra chromosome material. Dierssen presented her recent work using single nucleus RNA sequencing (snRNA-seq), which is a technique used to analyze the gene expression profiles of individual cells. Unlike traditional RNA sequencing (bulk RNA-seq), which measures the average gene expression across many cells, sn RNA-seq enables researchers to look at the transcriptional activity of individual cells, providing much more detailed and heterogeneous information about cell populations. Dierssen presented evidence showing that the impact of trisomy is highly cell-type specific and that the mechanisms regulating gene expression are highly cell-type specific, and thus, the full complexity of gene deregulation in DS is missed in bulk RNA-seq experiments. Her pioneering work using Genome Architecture Mapping (GAM) from CA1 pyramidal neurons of trisomic mice showed many altered 3D genome structures in the trisomic hippocampus. For example, loci of genes heavily downregulated in CA1 trisomic pyramidal neurons, like *Soga3 or Ptprk*, located close to each other in mouse chromosome 10, featured in extensively rewired 3D genome structures.

Evan E. Eichler (USA) presented the Global Down Syndrome Foundation Plenary lecture. Eichler discussed his most recent work sequencing diverse human genomes using ultra-long and high-fidelity, long-read sequencing technologies to fully phase and assemble diploid genomes with and without parental data. This technology allows sequencing to resolve most structural variants, including copy-number variants, irrespective of size—the vast majority of which are not routinely characterized by short-read sequencing. Eichler described methods using the strengths of two complementary long-read sequencing technologies to construct nearly complete telomere-to-telomere genomes where maternal and paternal complements were fully reconstructed. Their data included the resolution of telomeres, centromeres, and segmental duplications, allowing the variation of these regions to be investigated and their potential to contribute to disease and disease susceptibility. Assembly based variant discovery has the potential to provide a complete understanding of human genetic variation at every level and, Eichler predicts, will be the future of genetic- and clinical-based research.

### Epigenetic Changes in DS

Epigenetic studies on DS have gained attention as researchers explore gene expression modifications that influence observed phenotypes and health outcomes. Adam de Smith (USA) described recent epidemiological studies using neonatal dried bloodspot samples. Using DNA methylation array data, they discovered that newborns with DS have accelerated epigenetic aging in blood cells, with implications for the pathophysiology of immunosenescence and other aging-related traits. Finally, he discussed ongoing research investigating the genetic risk factors for leukemia in children with DS.

Conclusions: snRNA-seq provides a high-resolution view of gene expression at the level of individual cells, uncovering heterogeneity within specific cell populations. Long-read sequencing technologies represent a major leap forward in studies of complex genomes, detecting structural variants, and understanding gene expression at a detailed level. As technology continues to evolve, it is expected to become an increasingly important tool for DS research and clinical applications.

## Cellular and Molecular Mechanisms in DS

### Chromosome Engineering to Understand DS Phenotypes

Elizabeth Fisher (UK) gave a plenary lecture supported by the European Molecular Biology Organisation (EMBO). She described EMBO's origins, purpose, and functions and how they are relevant to us in T21RS. Fisher went on to consider what the future may look like for research into trisomy 21. She then presented some recent and largely unpublished results produced jointly by her lab and that of Victor Tybulewicz (UK) regarding how they have used mouse genetics, including a chromosome-engineered mapping panel, which was constructed by engineering and selecting specific chromosomal regions to create a library of modified chromosomes that can be used to identify and characterize genes associated with particular DS-related phenotypes. This includes novel findings for heart defects, craniofacial phenotypes, and—from the group of Steve Brown (UK) otitis media. Frances Wiseman (UK) used the same mapping panel to investigate genes on Hsa21 that modify the appearance of Alzheimer's changes in DS. Fisher presented ways of working with their mouse models to investigate the GABA-related phenotype found in the Dp1Tyb strain of mice, taking several different approaches from a group of collaborators. Fisher ended the talk by saying that the T21RS Preclinical Committee is providing accessible information on mouse models, cell models, and a potentially new tissue database of use to all researchers via the T21RS Preclinical Committee webpages.

### Intracellular Trafficking and Extracellular Vesicle Release in DS

This session discussed relevant findings about altered intracellular trafficking, which refers to the movement of molecules, such as proteins, lipids, and other cargo, within a cell. One key component involved in intracellular trafficking is the endosomal system, where endosomes play a central role in sorting, processing, and directing cargo to different destinations within the cell, including lysosomes, the plasma membrane, and other biologically active compartments within cells. The process of intracellular trafficking through the endosomal system leads to the release of extracellular vesicles (EVs), which are small vesicles that are secreted into the extracellular space. EVs have a potential use to measure disease biomarkers (Hamlett et al., 2018) and also provide new insights into cellular biology.

Marie-Claude Potier (France) interrogated the role of increased levels of APP (Amyloid Precursor Protein) in morphological aberrations in the endosomal compartment through the comparison of cortical pyramidal neurons obtained from postmortem brains of individuals with sporadic Alzheimer’s disease (AD), DS, and rare familial cases of AD [due to genomic duplication of the APP locus (*APP*dup)], as well as dermal fibroblasts derived from AD patients. Her group identified the increased density of normal-sized early endosomes in AD, DS and *APP*dup brain tissue compared to controls. These findings were recapitulated in AD-D fibroblasts and positively correlated with cognitive decline (Xicota et al., 2023). This work further implicates dysregulation in the endosomal compartment in peripheral cells, induced by the increased APP dosage, as a potential biomarker.

Xu-Qiao Chen (USA) discussed the causative link between increased levels of CTF (C-terminal fragment), a proteolytic product of APP, and the aberrant early endosomal compartment resulting in deficient retrograde axonal transport in DS. Using the Ts65Dn mouse model, Chen and his colleagues demonstrated that Posiphen, a small molecule compound, reduced the levels of APP and its fragments, rescued the enlargement of early endosomes and the activation of Rab5-mediated pathway, and restored normal neurotrophin signaling in the mouse brains. These studies further implicate increased dosage of APP as a key feature in the development of AD pathology in DS.

Ann-Charlotte (Lotta) Granholm (USA) presented how neuron-derived EVs (NDEVs), derived from blood samples of individuals with DS, spread different isoforms of phosphorylated tau (p-tau) to neuron and glia cells. By injecting NDEVs into hippocampi of adult wildtype mice, her group demonstrated that while p-(S396)-tau isoform was absorbed by both neurons and glial cells and involved white matter tracts, p-(T231)-tau isoform was primarily uptaken by neurons and was not detected in the white matter. This evidence suggested that seeding properties are distinct in different tau isoforms as NDEVs propagate them in the extracellular space.

Efrat Levy (USA) described a method her group developed for isolating EVS from brain tissue. This technology was applied to investigate the release of exosomes in the Ts2 mouse model. Her group found upregulated exosome secretion and enhanced generation of intraluminal vesicles in human and mouse DS brains and DS fibroblasts accompanied by increased levels of CD63, involved in exosome biogenesis. Silencing of CD63 inhibited EV secretion and worsened endosomal pathology, supporting the hypothesis that upregulated secretion of EVs in DS may actually relieve endosomal dysfunction.

In another study, Levy described that the number of EVs derived from mitochondria (called mitovesicles) is higher and that their composition is altered in the brain of the Ts[Rb(12.17(16))]2Cje mouse model of DS and individuals with DS compared with age-matched diploid controls. Additionally, human fibroblasts collected from individuals with DS, treated with antimycin-A (an inhibitor of the mitochondrial respiratory chain), release mitovesicles at higher rates than control euploid cells. Levy inferred that brain cells constitutively secrete mitochondrial vesicles more frequently in DS than in euploid individuals and that this pathway is altered under conditions where mitochondrial dysfunction occurs, including DS.

EVs are a promising non-invasive biomarker for early AD detection among individuals with DS. Aurélie Ledreux (USA) analyzed AD-related biomarkers [tau, p-tau, amyloid-beta (Aβ) peptides Aβ 1–40, Aβ1-42, and neurofilament-light] and neuroinflammatory biomarkers in NDEVs obtained from plasma of individuals with DS and correlated them with cognitive measures to investigate if they would predict disease trajectory and age of dementia onset. Ledreux proposed that neuron-derived extracellular vesicles present opportunities for biomarker discovery and potential early detection of AD in DS.

EVs provide novel snapshots of insulin signaling with DS. The alteration of the insulin signaling/mTOR (mammalian target of rapamycin) pathways represents an early event in the DS brain and likely contributes to cerebral dysfunctions and intellectual disability. Eugenio Barrone (Italy) reported that plasma-resident NDEVs isolated from children with DS can be characterized by a significant increase of pIRS1-(Ser636), a marker of insulin resistance, pIRS1-(Ser636), pAkt-(Ser473), pBAD-(Ser136), pmTOR-(Ser2448), pPTEN-(Ser380).

Conclusions: Elevated Aβ production in DS is linked to defective trafficking in the endosomal system, which contributes to neurodegenerative changes. The endosomal system plays an important role in cellular function, and its dysregulation in DS also contributes to enhanced EV production. EVs convey biomarkers of mitochondrial dysfunction, insulin signaling and pathological stress markers associated with AD. Understanding these mechanisms better may open up new avenues for treating the cognitive and neurodegenerative challenges faced by individuals with DS.

### Immune Dysregulation in DS

People with DS experience cognitive impairment, atypical morphogenesis, and inflammation. Although immune dysregulation is a hallmark of DS, the extent, etiology, and impact of co-occurring conditions in DS are still under investigation.

#### Inflammaging in DS

In this symposium, Bernard Khor (USA) presented on inflammaging in DS using deep immunophenotyping via mass cytometry, an advanced technique that allows for the detailed analysis of cellular phenotypes and functions by measuring multiple protein markers on individual cells. Khor demonstrated that many aspects of the global immune dysregulation characteristic of people with DS reflect advanced immune aging, or "inflammaging," in the absence of the co-occurring condition of autoimmunity by implementing a new analytical software (Lambert et al., 2022). To efficiently analyze these data, his team trained linear models of aging (i.e., "immune clocks") on peripheral blood samples from control disomic (D21) participants and then queried T21 blood samples for immune phenotypes associated with age. Through these efforts, people with DS were determined to experience advanced immune aging relative to a D21 cohort ~ 20 years their senior. They also shared many immune phenotypes with a separate D21 cohort bearing a chronic inflammatory autoimmune condition.

#### Interferon Response in Individuals with DS

Individuals with DS display a hyperactive interferon response, or interferonopathy, that involves overactivation of the type I interferon (IFN) system which leads to chronic inflammation and tissue damage. This response is likely driven by the increased dosage of four interferon receptors on Hsa21. This dysregulated interferon signaling, or interferonopathy, is likely to have important implications for the altered risk spectrum of co-occurring conditions in those with DS, especially immune-related disorders. Matthew Galbraith (USA) narrated how the Linda Crnic Institute Human Trisome Project collects clinical data and biospecimens from individuals with DS and euploid controls. They have generated matched 'omics datasets for ~ 400 participants. Using these datasets, they identified multi-omic signatures and co-occurring conditions associated with increased levels of interferon signaling in individuals with DS.

Katherine A. Waugh (USA) demonstrated that triplication of the IFN receptor (*IFNR*) gene cluster on Hsa21 potentiates multiple hallmarks of DS. Overexpression of these four *IFNRs* is associated with IFN hyperactivity and inflammation among whole blood transcriptomes from a large cohort of people with DS. To define the direct contribution of this locus to DS phenotypes, her team used genome editing to correct its copy-number in a mouse model of DS, which strikingly normalized lethal antiviral responses, prevented heart malformations, ameliorated developmental delays, improved cognition, and attenuated craniofacial anomalies. Therefore, an increased dosage of the *IFNR* gene cluster is necessary for increased severity and penetrance of major traits associated with DS, further supporting the notion that T21 elicits an interferonopathy amenable to therapeutic intervention. (Waugh et al., 2023).

Faycal Guedj (USA) presented a compelling case for broadly targeting immune system dysregulation and neuroinflammation in DS via prenatal therapy, as atypical brain development occurs in human fetuses with T21 as early as the second trimester. Towards this end, Guedj used preclinical models of DS to determine the safety and efficacy of apigenin, a flavonoid found in various plants, including chamomile. There was no significant increase in birth defects or pup deaths when apigenin was delivered prenatally to a mouse model of DS via food fed to pregnant dams throughout the life of progeny. There was, however, improvement in developmental milestone achievement timelines in neonates and spatial olfactory memory defects in adults. Of note, improvements mediated in a mouse model of DS by apigenin for exploratory behavior and long-term hippocampal memory were most prominent in males. Plausible mechanisms of action for apigenin improvement of cognitive and behavioral anomalies associated with DS that Guedj is currently pursuing include pro- and anti-inflammatory cytokines and neurotrophic factors, NFκB signaling cascades, and oxidative stress, particularly at the maternal–fetal interface. These data strengthen and expand on a growing body of literature that suggests that early treatment with broad anti-inflammatory agents may aid the neurodevelopment and cognitive function of individuals with DS.

*Conclusions*: Individuals with DS have a unique immune profile characterized by an increased interferon response. Understanding the role of interferonopathy in DS has opened avenues for potential therapeutic interventions. Targeting immune system dysregulation with compounds like apigenin could mitigate some of the immune-related and neurodevelopmental challenges seen in individuals with DS.

### Mitochondrial Dysfunction in DS

Several studies demonstrated that in people with DS, there are altered pathways involving key cell processes, such as mitochondrial metabolism (Caracausi et al., 2018; Izzo et al., 2018; Valenti et al., 2014) and one-carbon metabolism (Antonaros et al., 2021; Orozco et al., 2019). The one-carbon cycle (also known as the folate cycle) is a crucial biochemical pathway that involves the transfer of single-carbon units among molecules, which is essential for DNA synthesis, amino acid metabolism, and methylation reactions. Mitochondrial alterations can significantly impact the one-carbon (1C) cycle, as mitochondria play a critical role in supporting several reactions in this cycle, especially those involving folate and methionine metabolism. Interestingly, all these altered pathways seem to be involved in cognitive decline, thus highlighting the need to understand the crosstalk among metabolic and cognitive abnormalities.

Francesca Antonaros (Italy) studied one-carbon metabolism in DS using enzyme-linked immunosorbent assays to identify the concentration of 5 different intermediates of the one-carbon cycle namely tetrahydrofolate (THF), 5-methyl-THF, 5-formyl-THF,* S*-adenosyl-homocysteine (SAH) and* S*-adenosyl methionine (SAM) in plasma samples from 164 individuals with DS and 54 euploid individuals. Results highlight specific alterations of the one-carbon cycle, suggesting that the reaction in which homocysteine is transformed into methionine and 5-methyl-THF is converted to THF may be affected. Moreover, the 5-methyl-THF is the only folate form able to cross the blood–brain barrier because (1) it is the most reduced folate form, (2) it is well absorbed in the intestinal tract, and (3) additional enzymatic steps do not influence its bioavailability.

Antonella Izzo (Italy) investigated the role of peroxisome proliferator-activated receptor-gamma coactivator 1-alpha (PGC-1α), a master regulator of mitochondrial biogenesis and ATP production and related pathways in trisomic cells. Transcriptome analysis of human hearts and brains showed PGC-1α among the downregulated genes in the human brain and heart with trisomy 21, suggesting that this might cause mitochondrial dysfunction. More than one gene on Hsa21 can regulate PGC-1α expression. Izzo identified the corepressor NRIP1, which is upregulated in trisomic cells due to gene dosage. Knockdown of NRIP1 by siRNA can restore the level and function of PGC-1α protein and counteract mitochondrial dysfunction in trisomic fibroblasts. A similar result can be achieved using the drug metformin, which is known to activate PGC-1α transcription through AMPK and SIRT1. Moreover, Izzo has shown that mitochondrial dysfunction is already present in the early stages of differentiation of iPSCs from DS subjects into neuronal cells. Further studies are needed to assess whether mitochondrial dysfunction contributes to the alterations observed during neuronal cell differentiation (Mollo et al., 2020).

James Roede (USA) investigated basic mitochondrial biomarkers and functional metabolic assessments using extracellular flux analyses and stable isotope labeled (SIL) tracer metabolomics in human dermal fibroblast with trisomy 21 or normal karyotype. Results showed that the introduction of a stressor significantly impacted DS mitochondrial oxygen consumption rate because baseline assessments did not differ from controls. A 'developmental impact' on DS mitochondrial function included significant baseline differences in mitochondrial oxygen consumption in DS iPSCs compared to controls. Extracellular flux analyses of acidification rate did not reveal a significant glycolytic phenotype in DS fibroblasts; however, SIL metabolomic studies using labeled glucose and glutamine revealed altered central carbon metabolism in DS cells. These data indicate that aberrant central carbon metabolism is a candidate mechanism for stress-related mitochondrial dysfunction in DS (Anderson et al., 2021).

Conclusions: The therapeutic compound, 5-methyl-THF, has emerged as a strong candidate for clinical trials to restore regulation of the one-carbon cycle in T21, possibly improving the cognitive skills of individuals with DS (Vione et al., 2022). Mitochondrial dysfunction affects energy metabolism, disrupts the one-carbon cycle, and contributes to oxidative stress. Pharmacological or genetic enhancement of PGC-1α gene expression stimulates mitochondrial biogenesis and reduces oxidative stress in tissues affected by mitochondrial dysfunction.

## Alzheimer's Disease and Aging in DS

AD is a neurodegenerative disorder characterized by amyloid plaques mainly constituted of extracellular Aβ deposits in the brain parenchyma and intraneuronal neurofibrillary tangles consisting of hyperphosphorylated tau protein. The gene encoding for APP is triplicated in individuals with DS, leading to overproduction and abnormal accumulation of Aβ (Lott & Head, 2019). By 40 years of age, individuals with DS display full AD neuropathology, followed by AD dementia in their early 50 s (Iulita et al., 2022; Lott & Head, 2019). Thus, people with DS are at increased genetic risk of developing AD. In this session, AD neuropathology in DS, AD biomarkers, and clinical trials against AD in this population were discussed.

### DS and AD

Ira Lott (USA) discussed partial trisomy 21 and how the lack of overexpression of selective genes is helping researchers understand the role of genetics in the mechanism of AD. For example, a male with DS did not have an extra copy of the *APP* gene. Upon detailed examination over 7 years, the individual did not develop AD symptoms. Lott mentioned that individuals with DS afford an opportunity to understand lifespan changes that may determine healthy brain aging or a propensity towards dementia. In this way, brain development in people with DS can inform mainstream research into AD in the general population. Research studies have focused on factors responsible for AD beyond a single gene, including metabolic disturbances, brain inflammatory changes, and co-occurring medical conditions. People with DS stand at the threshold of clinical trial participation for the prevention or amelioration of AD. In this context, we need to understand research attitudes that may affect enrollment in these investigations. Standardized surveys are now underway. A compelling message that Lott left with the audience, including people with DS, self-advocates, and families, was this: "You teach us about other people with DS, you teach us about development and aging, and in the end, you teach us about ourselves."

### Cerebral Amyloid Angiopathy in DS and Familial Alzheimer's Disease with APP Duplication

Aβ peptides are often associated with amyloid plaques but can also be found deposited in blood vessel walls as cerebral amyloid angiopathy (CAA), a major cause of intracerebral hemorrhage. While the presence of amyloid plaques in *postmortem* brains is common to all AD cases, including sporadic, familial, and DS, CAA is a more prominent phenotype in familial cases with *APP* duplication (*APP*dup). The nature and mechanisms underlying these pathological and clinical differences remain unclear.

Amal Kasri (France) presented comparative neuropathological analyses of CAA in *APP*dup, DS, and other APP mutations. In *APP*dup cases, she described extensive CAA in all vessels, including capillaries, and fewer Aβ deposits in the parenchyma. Compared to *APP*dup, blood vessel amyloid deposits were less abundant in DS cases and scarce in sporadic AD cases. High levels of tau pathology were found in all cases with higher magnitude in DS with AD. To investigate the role of vascular endothelial cells and study the integrity of the blood–brain barrier in disease conditions, she derived endothelial cells derived from iPSC lines (iPSC-d-ECs) from patients. She identified changes in the morphology and tight junctions of iPSC-d-ECs with *APP*dup, suggesting intrinsic remodeling of ECs of the blood–brain barrier. She found that iPSC-d-EC secrete Aβ peptides that might accumulate in the extracellular space of blood vessels and contribute to the formation of vascular deposits. This study reveals pathophysiological mechanisms involved in specific Aβ production and deposition in the blood vessel wall of *APP*dup and DS.

Elena Camporesi (Sweden) showed qualitative and quantitative differences in Aβ peptides profile across the same diseases and cases as Amel Kasri. She analyzed the soluble and insoluble fractions by mass spectrometry using both MALDI and LC–MS following immunoprecipitation. The *APP*dup group showed the highest general abundance of Aβ with a specific increase of Aβ1-37/38/39/40 peptides and decrease of Aβ1-42. Interestingly, the Aβ2-x peptides were particularly increased. The DS with AD group also had increased Aβ1-37/38/39/40 peptides, while the DS without AD group had a pattern more similar to the control group. This study highlights the specific composition of Aβ peptides in *APP*dup cases associated with higher CAA pathology.

Paige Mumford (UK) presented neuropathological analyses of the brains of mouse and rat models of DS: AppNL-F model of amyloid pathology crossed with several DS mouse models (Tc1, Dp3Tyb, Dp(10)2Yey, Dp(17)3Yey) and new rat models with either two copies of humanized App with three copies of genes on rat chromosome 11 orthologous to Hsa21 (Dup(Rno11), including rat App, and Dup(Rno11)-APP-H3 with three copies of humanized Aβ sequence). Tc1*AppNL-F and Dp3Tyb*AppNL-F had reduced Aβ deposition compared to AppNL-F. Dup(Rno11)-APP-H3 rats had increased levels of FL-APP and insoluble and soluble Aβ1-40 and Aβ1-42, compared to APP-H and Dup(Rno11). No plaque deposition was seen in any genotypes. Further characterization of the rodent models will be necessary, but her results suggest that people with DS may be partly protected from their raised APP gene dose by the additional copy of other genes on the chromosome.

*Conclusions*: Endothelial cell secretion of Aβ is a potential source of AD pathological burden in blood vessel walls and may contribute to CAA. Specific Aβ peptide fragments are closely associated with CAA. Transgenic rodent models, harboring humanized forms of APP and rodent orthologs of trisomy 21, continue to be evaluated as models of amyloid pathology with DS.

### Interplay Between Inflammation, Neurodegeneration, and Aging in DS

Individuals with DS display a heightened neuroinflammatory profile (Flores-Aguilar et al., 2020; Martini et al., 2020; Wilcock et al., 2015). In this symposium, neuroinflammatory processes that can contribute to neurodegeneration and AD pathology were discussed.

Paula Araya (USA) presented a multi-omics analysis of neurodegeneration and neuroinflammation biomarkers, plasma proteomics, and immune profiling in a diverse cohort of 400 + research participants. They identified the depletion of insulin growth factor 1 (IGF1), a master regulator of growth and brain development, as the top biosignature associated with neurodegeneration in DS. Individuals with trisomy 21 display chronic IGF1 deficiency downstream of growth hormone production, associated with a specific inflammatory profile involving elevated TNFα. Shorter children with DS show more substantial IGF1 deficiency, elevated biomarkers of neurodegeneration, and increased prevalence of autism and other comorbidities. Altogether, these results indicate that inflammation-associated disruption of the IGF1 signaling pathway could contribute to stunted growth, neurodegeneration, and the appearance of key co-occurring conditions in DS.

Frances Wiseman (UK) described new bioinformatic approaches that identified genes on Hsa21 that could contribute to altered neuroinflammation in adults with AD (Braak stage VI) with and without trisomy 21 and aged-matched healthy aging controls. Analysis for the potential role of these candidate genes using a combination of in vivo studies and ex vivo organotypic slice cultures isolated from mouse models of DS (Dp1Tyb, Dp9Tyb, Dp3Tyb, Dp2Tyb, Dp10Yey and Dp17Yey) was performed. Observing that neuroinflammation (raised IL-β, TNF-α, and microgliosis in the hippocampus) occurs in an aging-dependent manner in the Dp1Tyb mouse model. Analysis of the smaller segmental duplication mouse models (Dp9Tyb, Dp2Tyb, Dp3Tyb) indicates that the altered neuroinflammation in the Dp1Tyb mouse is a multigenic phenotype. Moreover, altered Dp1Tyb neuroinflammation is not the result of enhanced sensitivity to inflammasome priming or activation. They revealed that an additional copy of the Dp2Tyb region (*Mis18a*-*Runx1*) was sufficient to cause a heightened response to interferon-β ex vivo. Overall, this indicates that interferon hypersensitivity may contribute to altered neuroinflammation in people with DS and that multiple Hsa21 genes are likely to contribute to the changes in neuroinflammation.

Peng Jiang (USA) explored the impact of trisomy 21 on microglia in DS brain development in DS with AD (DSAD). Using organoid and human-mouse chimeric brain models generated from human iPSCs highlighted that DS microglia show defective development and functions compared to control microglia. Moreover, in human-mouse microglial chimeric brains, DSAD human brain tissue-derived pathological tau induces senescence in DS microglia, recapitulating microglial responses in human AD and DSAD brain tissue. Importantly, inhibiting the expression of Hsa21-encoded type I interferon receptor genes (IFNARs) improves the defective DS microglia functions during brain development and prevents DS microglial senescence in response to pathological tau, suggesting that new therapeutic strategies targeting IFNARs could prevent human microglial senescence to potentially slow the progression of AD in DS.

*Conclusions*: In individuals with DS, neuroinflammation can contribute to neurodegeneration and other co-occurring conditions. Moreover, neuroinflammation in DS may be the result of multiple genes encoded in chromosome 21. By inhibiting interferon receptor genes, researchers were able to restore microglia functions.

### AD Biomarkers in DS

Neuroimaging with Positron Emission Tomography (PET) imaging and Magnetic Resonance Imaging (MRI) have become crucial tools in the clinic and are often correlated to other test indicators to enhance assessment, diagnosis, and monitoring of AD. Given the unique biology of people with DS and their high-risk for AD, this population provides an unparalleled opportunity to investigate and develop biomarkers of preclinical AD. Reliable biomarkers of preclinical AD enable new options for intervention and promote health for people with DS and in the general population. The Alzheimer Biomarker Consortium-DS (ABC-DS) is an ongoing, multisite study with the goals of identifying cognitive, blood, CSF, and neuroimaging biomarkers for predicting the onset and progression of AD in DS (Handen et al., 2020). Components of the ABC-DS study include investigations of lifestyle factors, neuroimaging biomarkers, and neuropathological features in DS related to AD (Handen et al., 2020). Other international initiatives to explore AD biomarkers in people with DS include the Down Alzheimer Barcelona Neuroimaging Initiative (DABNI). DABNI is a comprehensive biomarker program that includes MRI, amyloid PET, CSF, blood, genetic analyses, as well as sleep studies to unravel the mechanisms leading to AD dementia in adults with DS. The DABNI cohort includes over 700 participants to date and results from this cohort were discussed in the session “Conducting Alzheimer’s disease prevention trials in adults with DS” by Isabel Barroeta.

#### Weight Loss and AD Risk in DS

Unintentional weight loss may be predictive of the early AD risk for individuals with DS. Victoria Fleming (USA) presented a study examining the relationship between body mass index (BMI) and AD in DS. In midlife, a BMI over 25 is associated with an increased risk of AD, while unintentional weight loss or reduced BMI in older adults has been linked to cognitive decline and diagnosis of AD dementia. These findings have led many to view unintentional weight loss as part of preclinical AD. Using the Cued Recall Test (CRT) and the DS Mental Status Exam (DSMSE) as indices for cognitive functioning, models were examined to assess associations with PET-measured amyloid and tau burden. Results revealed that weight began to decline during the late 30 s, and adults with DS who were Aβ + evidenced greater weight loss across the time points (16–20 months) compared to the Aβ- group, after controlling for age. The presence of tau also revealed an association with BMI. A greater decline in the DSMSE was associated with greater weight loss in those who were Aβ + /tau-. These findings have implications for clinical AD interventions and suggest that weight loss could be an important early symptom of AD in DS.

#### Advanced MRI-Based Modeling of Structural Connectome and Prediction of PET-Measured Amyloid Burden

Stephanie Brown (UK) introduced the use of artificial intelligence to gain an understanding of how neuroimaging data can provide insight into changes in the brain related to cognitive and functional performance in adults with DS. Diffusion-weighted imaging and connectome modeling were analyzed to predict PET-measured brain amyloid plaque burden, baseline cognition and longitudinal cognitive change using support vector regression. The results demonstrated that graph theory metrics of node degree and strength based on the structural connectome are effective predictors of global amyloid burden. Also, revealing that the connection density of the structural network at baseline measures is a promising predictor of current cognitive performance. Further, the use of mixed effects models revealed the presence of PET tau was a significant independent predictor of cognitive and functional change. These findings contribute to an emerging framework in which the elevated risk of individuals with DS developing dementia involves mechanisms associated with both Aβ and tau aggregation, similar to processes found in the neurotypical population.

Jeremy Rouanet (USA) discussed neuropathological investigations of amyloid in postmortem brain tissue in DS. The regional progression of amyloid buildup in DS is largely similar to that of sporadic AD in the neurotypical population based on PET studies using the Pittsburgh Compound B (PiB) radiotracer. However, a unique pattern emerges in people with DS, where the earliest and strongest PiB retention is in the striatum, unlike sporadic AD. A fluorescent version of PiB, cyano-PiB (CN-PiB), was used to investigate PiB binding in DS postmortem brain tissue. Whole slide imaging and automated image analysis were then used to identify individual plaque-level information regarding load, size, and distribution of CN-PiB + plaques in DS. These ongoing studies will shed light on the neuropathology of CN-PiB binding in the striatum, and how it relates to patterns in the frontal cortex, and the progression of AD in DS.

*Conclusions*: Unintentional weight loss may be a useful bioindicator of early AD for individuals with DS. Advanced MRI modeling can predict PET-measured brain amyloid plaque burden, baseline cognition and longitudinal cognitive change. Studies of the PiB PET tracer revealed an early pathological signature in the striatum of individuals with DS compared to sporadic AD cases. Several ongoing biomarker and neuroimaging initiatives will accelerate innovation in DS-related AD assessment, diagnosis, and monitoring.

### Conducting AD Prevention Trials in Adults with DS

People with DS represent a priority population for preventive clinical trials (Fortea et al., 2021). Recent milestones in DS research include the effort of several international groups to map the natural history of AD through clinical, fluid and neuroimaging measures.(Carmona-Iragui et al., 2021; Fagan et al., 2021; Fortea et al., 2018b, 2020; Janelidze et al., 2022; Rafii et al., 2017). These studies showed that the trajectory of biomarker changes in DS resembles that of genetic and sporadic forms and that people with DS are willing and capable of undergoing all the procedures required in a clinical trial. However, important questions remain, including (1) Are people with DS involved in AD clinical trials? (2) Which are the best tools for patient selection, assessing target engagement and outcome measures? and (3) Are there harmonization efforts in place?

In this session, Michael Rafii (USA) reviewed the current state of clinical trials for AD in persons with DS, including completed, ongoing, and planned studies, as well as the concurrent development of therapeutics for other forms of AD (i.e., preclinical, prodromal, and autosomal dominant forms). Rafii discussed the progress made in disease-modifying treatments for AD, including the recent accelerated approval of aducanumab based on amyloid removal, as well as ongoing trials for the preclinical (asymptomatic) stage of sporadic AD with other anti-amyloid immunotherapeutics in the pipeline, i.e., lecanemab, donanemab, and gantenerumab. All utilize plasma biomarkers during screening prior to amyloid PET scan for eligibility. None of these clinical trials enrolled individuals with DS. Rafii further described how the AT(N) framework allowed the biological staging of AD, providing a path forward towards approval under the 2018 FDA Guidance defining 'Early AD.' The presentation highlighted that disease-modifying therapeutics are urgently needed for individuals with DS and that amyloid represents a prime target, with advances being made in sporadic and autosomal dominant forms. Finally, he described how the Alzheimer's Clinical Trials Consortium-DS (ACTC-DS) will help bring the latest therapeutics to the population with DS.

Asaad Baksh (UK) continued with a discussion of the barriers that have limited the involvement of people with DS in AD prevention trials. These include the limited research on which cognitive abilities are most sensitive to early AD-related impairments, the lack of standard endpoints to assess efficacy, and the limited research on the earliest age at which AD-related decline begins in this population. Baksh presented work from the LonDownS consortium (King's College London, UK), the Horizon 21 (H21) Consortium in Europe (Aschenbrenner et al., 2021; Firth et al., 2018; Hithersay et al., 2021) and with Washington University in St Louis, Missouri, USA. Results included a data-driven approach to identifying cognitive test items that are the most sensitive to detecting early cognitive change in adults with DS, validation of a dementia staging model and a novel approach to identifying the earliest age of change in cognition in order to guide optimal recruitment periods for clinical trials (Aschenbrenner et al., 2021; Firth et al., 2018; Hithersay et al., 2021). Sample size calculations have also shown that randomized clinical trials are feasible in DS (Aschenbrenner et al., 2021). Numerous research groups contribute to international harmonization collaborations to streamline data sharing and create the pipelines needed to conduct these trials in DS.

Isabel Barroeta (Spain) provided a thorough overview of the link between AD and DS, including recent work highlighting the association of this disease with mortality and life expectancy and a systematic review and meta-analysis revealing the conserved age at diagnosis in this population (Iulita et al., 2022). The presentation then focused on fluid (CSF, plasma) and imaging (MRI, PET) biomarkers of amyloidosis, tau pathology, and neurodegeneration and how they have shown excellent diagnostic performance in distinguishing symptomatic AD in adults with DS using data from the DABNI cohort (Carmona-Iragui et al., 2021; Fortea et al., 2018a, 2020). These biomarkers followed a similar temporality of changes with respect to sporadic and autosomal dominant forms. Barroeta further described how these different biomarkers enable the development and monitoring of effective disease-modifying therapies, as well as the identification of potential study participants most likely to respond to therapies. She described how to consider individual heterogeneity, such as variations in the localization of pathology at different stages, the level and type of glial reaction, and vascular or other co-pathologies, indicating that certain subgroups, potentially defined by biomarkers, might respond better to certain therapies.

Alberto Costa (USA) described how preclinical evidence from mouse models of DS (primarily Ts65Dn but also Ts1Cje) showed that the brain information pathway that uses the amino acid glutamate as its main neurotransmitter may be altered in persons with DS. This is the so-called glutamatergic hypothesis for DS. In particular, he presented data from a concluded phase II clinical trial of the FDA-approved AD drug memantine (Costa et al., 2022). In this trial, it was learned that memantine was well-tolerated by adolescents and young adults with DS and that, when given at the standard dose used to treat AD (20 mg P.O. per day), this medication did not enhance cognitive test scores from the study participants. However, post hoc analysis of the data indicated that higher-than-standard doses of memantine may produce cognitive enhancement in persons with DS (Costa et al., 2022). The Costa group plans to further confirm these findings in future studies.

*Conclusions*: Unprecedented research activity in biomarkers in DS is rapidly changing the clinical trial landscape in AD, which could facilitate the evaluation of disease-modifying therapies that will also benefit other genetic and sporadic populations. Indeed, the ultra-high-risk for AD in DS and the high diagnostic certainty for an underlying AD pathology in this population enables opportunities for early intervention that might be unfeasible in sporadic AD (André Strydom et al., 2018a, 2018b).

## Co-Occurring Conditions, Treatments, and Clinical Trials in DS

Co-occurring conditions are common in people with DS. This session focused on treatments and clinical trials in obstructive sleep apnea, immune system dysregulation, obesity, and psychiatric disorders, among others.

### Research Advances in Obstructive Sleep Apnea Treatment for People with DS

People with DS have a high prevalence of obstructive sleep apnea (OSA) throughout their lifetimes, with a negative impact on overall health (Gimenez et al., 2021). Detection, however, is too often overlooked and delayed. There is an imperative need for an early and effective OSA treatment to minimize these consequences. This session presented recent advances in OSA treatment with positive airway pressure (PAP) and hypoglossal nerve stimulator devices in children, adolescents and adults.

Ignacio Tapia (USA) discussed the relevance of OSA in children with DS, and provided an overview of available treatments with an emphasis on PAP (Xanthopoulos et al., 2022). Specifically, Tapia presented strategies to improve adherence to PAP and data on PAP adherence in children with DS. The latter showed improved adherence over 2 years, with more than 50% of children with DS using their PAP over 4 h more than 50% of the nights (Xanthopoulos et al., 2019).

Sandra Giménez (Spain) reviewed the pitfalls and controversies regarding the feasibility and adherence of PAP treatment in adults with DS. Despite the high prevalence of obstructive sleep apnea in adults with DS, PAP treatment is not regularly proposed since it is assumed by physicians and caregivers that patients will not tolerate the treatment. Giménez presented prospective data evidencing good PAP compliance after 3 years of follow-up, with the same habitual clinical PAP management criteria used in the euploid control population. The number of hours of PAP use at the first visit predicted long-term PAP compliance in both groups (Gimenez et al., 2022).

Brian Skotko (USA) presented the results from another OSA treatment modality with hypoglossal nerve stimulator devices. He presented recent prospective data of an ongoing single-group multicenter cohort study looking at the safety and efficacy of hypoglossal nerve stimulation in people with DS. The results demonstrated that the device was safely implanted for 42 adolescents who have DS and persistent severe OSA after adenotonsillectomy with PAP intolerance. There was an acceptable adverse event profile with high therapy response rates and quality of life improvement (Yu et al., 2022).

Daniel Combs (USA) presented on children with DS and OSA. Airway hypotonia is an important cause of OSA in people with DS and presents a unique opportunity for targeted intervention. The combination of atomoxetine and oxybutynin (ato-oxy) targets airway hypotonia in people with OSA. Data in adults without DS has shown ato-oxy appears safe and effective for treating OSA. Given that airway hypotonia is an important factor for OSA in people with DS, this combination may be particularly effective in people with DS. The combination of atomoxetine and oxybutynin (ato-oxy) is being investigated in an ongoing double-blind clinical trial of obstructive sleep apnea in children with DS. Preliminary results suggest that ato-oxy is typically safe in children with DS. The most common adverse events to date have been fatigue and irritability, which are known effects of atomoxetine.

Conclusions: Recent advancements in the treatment of OSA in individuals with DS highlight promising approaches, including PAP and hypoglossal nerve stimulation devices. PAP adherence has shown improvement in both children and adults with DS, demonstrating long-term benefits when properly managed. However, treatment adherence remains a challenge, especially in adults, where assumptions about tolerance often delay treatment. Additionally, the combination of atomoxetine and oxybutynin (ato-oxy) is being explored as a targeted intervention for airway hypotonia in children with DS, with preliminary results suggesting it may be a safe and effective treatment for OSA.

### Current and Future Directions in Interventional Clinical Trials to Improve Health Outcomes in DS

Given the distinct biology resulting from trisomy 21, the clinical management and treatment of co-occurring conditions in the population with DS requires special considerations. This symposium highlighted interventional clinical trials designed exclusively for people with DS.

Angela Rachubinski (USA) presented preliminary interim analysis results describing the use of the JAK-inhibitor, tofacitinib, to address the hyperactive JAK/STAT signaling and chronic dysregulation of the immune system seen in DS, which can manifest as immune-mediated skin conditions. Results from the first 10 participants indicate that tofacitinib is well-tolerated in people with DS, and both interferon scores and cytokine scores decreased after 16 weeks of treatment. Skin pathology showed improvement, especially in the treatment of alopecia areata. Additional research is required in individuals with DS and hidradenitis suppurativa. Preliminary results show improvements in quality of life, anxiety, depression, and spatial memory, with encouraging results in episodic and visuospatial memory tests.

Anna Esbensen (USA) described preliminary results of a clinical trial in children with DS and Attention Deficit Hyperactivity Disorder (ADHD) who are being treated with methylphenidate. Children with ADHD and DS are treated with stimulant medication at a much lower rate than in the typical population, despite its proven effectiveness for managing ADHD symptoms. The study design addresses potential reasons that stimulant medication may be under-utilized for children with DS, including (1) a rigorous definition of clinical features of ADHD in DS and (2) the short- and long-term data to be collected to address potential safety concerns, with particular attention to safety in the presence of congenital heart defects.

Conclusions: The use of the JAK-inhibitor tofacitinib has shown positive preliminary results in improving immune dysregulation and skin conditions, such as alopecia areata, while also enhancing quality of life, psychiatric disorders, and memory. Additionally, the potential reasons for underutilization of stimulant medications in DS were discussed. These studies highlight the need for tailored therapies to improve health outcomes in the DS population.

### Progress and Challenges in Therapy for Children with Trisomy 21 and Acute Leukemia

Children with DS have a tenfold increased risk of developing acute lymphoblastic leukemia (ALL) and demonstrate a very different spectrum of somatic alterations, including cytokine receptor-like factor 2 rearrangements (CRLF2-R), associated with reduced B-cell differentiation (Junco et al., 2022), in approximately 50% of cases, compared to only 5–10% of non-DS ALL cases. Although the increased risk of leukemia has been recognized for decades, the underlying biological basis remains unclear.

Rabin (USA) discussed somatic features defined by cytogenetics and whole genome and transcriptome sequencing, germline variants associated with increased susceptibility to ALL, and the interplay between other DS-related phenotypes (e.g., structural birth defects) and ALL susceptibility. Further, Rabin reviewed the 20-fold increased risk of acute leukemia in children with DS, which comprises approximately 2% of childhood acute ALL and 10% of childhood acute myeloid leukemia (AML). She presented some hypotheses for the increased risk of leukemia, including an increased dosage of genes on Hsa21 and differences in epigenetic regulation, hematopoiesis, immune function, and various cellular pathways. Rabin reviewed the distinct spectrum of genomic alterations in DS ALL, including the increased frequency of *CRLF2* rearrangements and *JAK* mutations and decreased frequency of favorable and unfavorable recurrent alterations observed in non-DS ALL. Rabin also discussed the clinical features of transient abnormal myelopoiesis (TAM), a unique megakaryoblastic proliferative disease observed in the fetal and neonatal period in newborns with DS, which demonstrates highly variable severity, spontaneous resolution within several weeks to months, an association with somatic *GATA1* mutations, and an increased risk of subsequent development of myeloid leukemia of DS (ML-DS).

Johann Hitzler (Canada) reviewed the 150-fold increased incidence of ML-DS, the younger age at diagnosis, and the specific disease mechanism driven by somatic mutations in the gene coding for the hematopoietic transcription factor *GATA1*. He presented the overall favorable survival prognosis of ML-DS, achieved in contemporary clinical trials by multiple study groups (event-free survival 90%). Tracing the trajectory of trial design for ML-DS, Hitzler illustrated the early priority of reducing treatment-related mortality by designing DS-specific clinical trials with a progressive reduction of treatment intensity. Hitzler highlighted that the most recent study by the Children's Oncology Group (AAML1531) reached the limit of this approach when further reduction of treatment intensity resulted in an uptick in relapse events. He showed that compared to children without DS, the prognosis of myeloid leukemia was significantly better in children with DS, but that in the event of a relapse, the probability of survival was significantly worse. Cytogenetics, targeted sequencing, and error-corrected sequencing of patient-specific *GATA1* mutations to measure early response to treatment are being evaluated as prognostic markers. Reduction of relapse events was described as the major goal of future trial design in ML-DS. Finally, clinical management of TAM was reviewed, including its almost universal spontaneous resolution, the clinical indication for low-dose chemotherapy in a subset of patients with severe disease, and candidate prognostic markers, such as delayed resolution of TAM, that may predict the progression to ML-DS that occurs in 20% of children with TAM.

Amanda Li (Canada) reviewed the 20-fold increased risk of ALL in children with DS. Patients with DS and ALL have historically shown inferior responses to therapy and higher rates of relapse compared with patients with DS. Moreover, patients with DS and ALL show a unique vulnerability to treatment-related toxicity, primarily infections, which are the leading cause of treatment-related mortality in this population. They are also three times as likely to experience hyperglycemia and four times as likely to experience mucositis associated with chemotherapy compared with patients without DS. A subset of patients with DS with additional high-risk features have been shown to have an increased risk of seizures during treatment of ALL. Hence, patients with DS receiving treatment for ALL have shown benefit from increased supportive care measures during treatment, including empiric anti-microbial prophylaxis and closer monitoring in a hospital setting. For patients who experience ALL relapse, the ability to safely deliver intensive chemotherapy at the intensity required to eradicate the disease remains challenging. Therefore, novel targeted and immune-based therapies such as blinatumomab and inotuzumab antibody therapy and chimeric antigen receptor (CAR) T-cell therapies are being explored in this patient group to achieve a cure without unacceptable toxicity.

Conclusions: Children with DS are at a significantly higher risk of developing acute leukemia, with distinct somatic alterations, such as CRLF2 rearrangements, contributing to the disease. While treatments for ML-DS show favorable survival rates, the challenge remains in reducing relapse events and treatment-related toxicity. For DS-related ALL, supportive care is crucial to mitigate treatment-related complications, and novel therapies like targeted antibody treatments and CAR T-cell therapies are being explored to improve outcomes with lower toxicity.

### Obesity and Metabolic Multi-Morbidities in DS

Obesity is common in DS and is associated with additional metabolic multi-morbidities. Understanding the pathways and mechanisms driving obesity in DS and its multi-morbidities is of major importance for the treatment of DS individuals with such conditions. As obesity is the result of complex relationships between genetic, socioeconomic, and cultural influences, to truly understand the contribution of each component requires new approaches to integrate molecular (multi-OMICS) preclinical (cellular and animal) and clinical datasets and metadata (environment, patient history). In this symposium, work was presented on the Gene Overdosage and comorbidities during the early lifetime in DS (GO-DS21) pan-European consortium, consisting of 12 partners from 6 European countries coordinated by Yann Herault (France).

Sarah Pape (UK) presented a study of national UK data spanning 3 decades, with 9917 individuals with DS and 38,266 euploid controls. Overall incidence rates of diabetes were 3.7 times higher in people with DS who were also diagnosed at an earlier age than controls. BMI was also higher in DS compared to control with peak values observed at an earlier age. The talk concluded that there were significant differences in the metabolic profiles of people with DS compared to controls, with shifts in age-related comorbidity patterns. This talk highlighted the power of large national electronic health records and datasets that can be utilized to decipher the natural history of complex comorbid phenotypes such as those seen in DS.

Maria Martinez de Lagran (Spain) presented work on *Dyrk1A* (a gene triplicated in DS) and how under and over-expression of *Dyrk1A* in rodent models altered palatable food overconsumption through dopaminergic mechanisms. The team fed mice high-fat or chocolate diets and used chemogenetic tools to dissect the neuronal circuitry involved in DS-associated hedonic overeating. They concluded that *Dyrk1A* was involved in hedonic overeating, specifically upon exposure to a chocolate diet. In addition, *Dyrk1A* regulates compulsive feeding behavior and cognitive inflexibility. They concluded that *Dyrk1A* may be a good candidate gene for targeting obesity in DS individuals. The talk expanded the role of *Dyrk1A* in DS and highlighted progress in the use of mouse models and state-of-the-art chemogenetic techniques to decipher neuronal circuits associated with feeding behavior in DS.

Pietro Barbiero (UK) gave an overview of the integration of bioinformatics and the use of artificial intelligence (AI) in complex diseases such as DS, data integration, analysis, and visualization. He discussed the basic concepts, the direction of handling counterfactuals, explainability, and interpretability using deep learning in medicine. He also discussed concepts around using digital twins in medicine. The conclusion of the talk is that such techniques are here to stay and are a powerful approach to medicine. Collectively, the talks demonstrate the understanding that a coordinated approach using the latest AI techniques is required to truly integrate datasets derived from both humans and rodents to decipher the importance of gene overdosage and comorbidities during the early lifetime of people with DS.

Conclusions: Individuals with DS may have a higher incidence an earlier onset of diabetes and higher BMI compared to those without DS. Research on Dyrk1A in rodent models has shown its involvement in overeating behaviors, highlighting it as a potential therapeutic target. The integration of advanced techniques like AI is crucial for understanding the complex interplay of genetics and environmental factors in DS-related obesity and other co-occurring conditions.

## COVID-19 in DS

People with DS are at increased risk of infections with respiratory complications and immune system dysregulation (Illouz et al., 2021a, 2021b; Illouz et al., 2021a, 2021b). They also present with multiple co-occurring conditions that are associated with poorer COVID-19 prognosis than in the general population (Illouz et al., 2021a, 2021b; Illouz et al., 2021a, 2021b). The T21RS has constituted a global task force of researchers from different nations to study the effects of COVID-19 infection, its clinical presentations, treatment, and vaccination. This session focused on different aspects of COVID-19 infection among individuals with DS.

Mara Dierssen (Spain) presented the outcome of a network analysis that reveals the interplay between trisomy 21 and SARS-cov2 infection. Her team detected COVID-19 protective and risk factors among Hsa21 genes and interactors and DS-deregulated genes that might affect the susceptibility of individuals with DS both at the infection stage and in the progression to acute respiratory distress syndrome.

Alberto Costa (USA) narrated an overview of the global survey conducted by the T21RS COVID-19 DS task force. The data from individuals with DS from more than 10 different countries and more than 2000 unique cases that enrolled since April 2020 exhibited a higher risk of COVID-19 infections and significantly higher rates of medical complications and mortality, especially from age 40 and above. Further, the study found that children with DS are at increased risk for more severe presentations of COVID-19. A study on vaccination for individuals with DS revealed that COVID-19 vaccines are safe for individuals with DS and effective at minimizing breakthrough infections and mild disease outcomes among fully vaccinated individuals with DS.

Angelo Carfi (Italy) compared antibody responses between 42 subjects with DS and age- and sex-matched comparison group of healthy healthcare workers after SARS-CoV2 vaccination with the standard regimen of BNT162b2 mRNA COVID-19. They observed significantly different antibody responses at all time points after vaccination and a significantly different course of decline in antibody titers between the two groups. They concluded that individuals with DS have a valid antibody response to SARS-CoV2 vaccination, but the response is lower than that of subjects in the control group.

Pinku Halder (India) described the outcome of the psycho-social impact of the COVID-19 pandemic on individuals with DS and their family members. He reported that children with DS experienced more disturbed home life, emotional distress, and longer quarantine during the pandemic in comparison to euploid children from the general population. On the contrary, the cancellation of social programs was more frequent in neurotypical control children than in children with DS. Halder found more adverse effects of the pandemic, such as emotional health and medical care, in families with a member with DS. Moreover, parents and caregivers had a longer quarantine period than control families. The home life of control families was more disturbed by frequent conflicts with spouses or partners.

Sujay Ghosh (India) presented the results of a comparative study on comorbidities, clinical presentation, treatment, disease severity and outcome among COVID-19-infected individuals with DS in India a low to middle-income country) and high-income countries (USA, UK, France and Spain). Compared to high-income countries, individuals with DS from India exhibited more frequent occurrences of comorbid conditions, more frequent hospitalization and critical care support provided to the patients, more frequent use of antibiotics, and more severe outcomes of the COVID-19 infection. Ghosh inferred that observed differences may arise from socioeconomic and medical practice differences between India and high-income countries. This observation may be helpful in resource and medical support allocation to the countries in the next global health crisis.

Conclusions: Network analysis identified protective and risk factors for COVID-19 related to T21 genes. The T21RS global survey showed a higher risk of COVID-19 complications and mortality in DS, particularly in adults over 40, but found vaccines safe and effective. Studies also revealed lower antibody responses in DS individuals post-vaccination compared to controls. Additionally, the pandemic had a significant psycho-social impact on individuals with DS and their families. Individuals with DS in low-income countries, like India, faced more severe COVID-19 outcomes compared to those in high-income countries.

## Machine Learning and Neurodevelopmental Disorders

This session discussed the “lessons learned” from the translational failure of prior treatment trials in individuals with DS despite promising preclinical studies (Lee et al., 2020; Rueda et al., 2020). The session emphasized that this translational gap extends to other neurodevelopmental disorders (NDDs), including autism spectrum disorder (ASD), Fragile X (FXS), and CDKL5 deficiency disorder (CDD) (Berry-Kravis, 2022; Diaz-Caneja et al., 2021; Tranfaglia et al., 2019).

The session started with an opening presentation by Faycal Guedj (USA) proposing a new research vision entitled “Translation 360” to increase the likelihood of developing safe and effective therapeutic interventions in the future (Lee et al., 2020). Translation 360 is a "Transversal" research pipeline that emphasizes the development of reliable and translational endpoints, druggable targets, and biomarkers through multicenter clinical research studies that use standardized outcome measures in individuals with NDDs. In parallel to developing these endpoints, druggable targets, and biomarkers, it is critical to combine patient-derived iPSC cell models and mouse models that replicate the human genotype, karyotype, and phenotype prior to engaging in timely and costly treatment trials. This vision requires the full integration of clinical and preclinical research data using novel, unbiased computational approaches and machine learning algorithms. Finally, future treatments should be tested and replicated in both human iPSC-derived cell models and mouse models for safety and efficacy using study designs that systematically integrate random sampling and blinding like what is routinely used in human clinical trials to reduce bias and increase the reliability in preclinical treatment studies.

Despite extensive endeavors to uncover the etiology of NDDs, their intricate and multifaceted nature presents significant challenges in pinpointing the core molecular networks consistently impacted in preclinical models and humans affected by these conditions. In recent years, we have witnessed significant strides toward using unbiased multi-omics approaches and machine learning to identify molecular networks affected by different human conditions and potentially targeted in forthcoming therapeutic interventions (Yu et al., 2020) (Ferraro et al., 2023; Wang et al., 2021). However, pivotal questions persist, including: (1) can machine learning effectively bridge the translation gap? and (2) which machine learning models hold the key to achieving this transformative goal?

To address these questions, Aris Persidis (Biovista CEO) argued in his presentation that machine learning is domain-specific and that, in contrast, many machine learning applications in diagnostics, self-driving cars and games where algorithms use training data to extract patterns that are already present (known), different machine learning models should be built specifically for biomedical research to extract possible answers that remain unknown. In this context, Biovista Inc. has developed a new supervised Augmented Intelligence model (Project Prodigy) that integrates large amounts of clinical, preclinical and pharmacology/toxicology data to develop new hypotheses of disease etiology and repurpose small molecules (Andronis et al., 2011; Persidis et al., 2004). Using FXS as an example, this new machine learning model gathered and organized 2708 genes studied in connection to the condition, 453 pathways, 18 post-translational modifications, 212 cell types, 374 drugs, and multiple other types of data from the approximately 9400 published papers in PubMed. This new intense data-driven approach could synthesize previous studies and combine them with novel omics datasets to have a holistic view of mechanisms of action, translation paths, and possible interventions for NDDs.

Massimiliano Bianchi (CEO of Ulysses Neuroscience) discussed the translational work that his team, as well as other pharmaceutical companies (e.g., Roche, Ultragenyx, Takeda), are doing to develop biomarkers and treatments for CDD resulting from mutations in the *CDKL5* gene and affecting 1 in 40,000–60,000 newborns with severe comorbidities, including developmental delay, epilepsy, intellectual disability, dysphagia, dystonia and breathing disturbances (Leonard et al., 2021). Bianchi's team is actively investigating the link between defects in α-tubulin post-translational modifications and abnormal brain development and cognition in Cdkl5-KO mice and their potential use as biomarkers for CDD. Through the integration of preclinical data and clinical data from multicenter clinical studies, his team demonstrated that the ratio of acetylated/total tubulin is consistently increased in the brain and plasma of Cdkl5-KO mice as well as in the plasma of CDD patients, suggesting that plasma Acetylated/Total tubulin is a reliable biomarker for CDD. His team also used both pharmacological (pregnenolone) and gene therapy approaches (Cdkl5 AVV) to rescue the ratio of acetylated/total tubulin ratio in the Cdk5l-KO plasma and brain (Barbiero et al., 2022a, 2022b; Barbiero et al., 2022a, 2022b). Ulysses Neuroscience's long-term goal is to translate these promising preclinical findings into effective therapies for patients with CDD.

Conclusions: This session emphasized the integration of preclinical and clinical data using machine learning algorithms to advance translational research. Further, progress was made in developing biomarkers and treatments for CDD, including acetylated/total tubulin as a potential biomarker.

## Language Outcome Measures for Clinical Trials: from the Prelinguistic Period into the Early Adulthood

Language impairments are frequent, severe, and of prognostic value in individuals with DS. Consequently, clinical trials targeting language and communication are needed for this population. Unfortunately, the evaluation of the efficacy of treatments continues to be hindered by a lack of appropriate outcome measures (Esbensen et al., 2017). In this symposium, researchers presented data on three different research programs focused on developing and validating outcome measures derived from analyses of natural communication samples.

Angela John Thurman (USA) reviewed the methodological challenges associated with characterizing language progress in prelinguistic and early linguistic skills and provided preliminary data on the utility of various outcome measures derived from play-based communication assessments for children with DS between 2 and 8 years of age. The multisite project uses natural communication sampling, which entails recording and analyzing the frequency and quality of communication of young children with DS in various semi-structured play activities.

Leonard Abbeduto (USA) described an ongoing multisite project documenting the utility of expressive language sampling (ELS) as a source of psychometrically sound outcome measures for treatment studies and its correlation with functional outcomes. ELS entails collecting samples of spoken language from participants interacting with an examiner in naturalistic yet standardized interactions involving individuals with DS, ages 6–23 years (Abbeduto et al., 2020; Thurman et al., 2021).

Laura del Hoyo Soriano (USA) presented results from a pilot study that examined the feasibility of teaching native English-speaking parents and native Spanish-speaking parents how to administer ELS procedures to their sons and daughters with autism (between ages 6 and 21) at home through telehealth-delivered procedures. Most of the parents who participated in this project were able to learn the ELS procedures to the target level of fidelity, with the procedures being feasible for the majority of the participants with autism and with no practice effects and appropriate test–retest reliability (Del Hoyo Soriano et al., 2022; Soriano et al., 2021). This small-scale pilot project is promising for the DS population.

Conclusions: This symposium highlighted three research programs focused on developing reliable language outcome measures. The use of play-based communication assessments for young children with DS, ELS for individuals aged 6–23 years, and teaching parents to administer ELS procedures via telehealth. Collectively, these three projects offer a new set of tools for measuring language and communication improvements in treatment studies for DS.

## Biobanks and Biomarkers Offer Support for Translational Research in Down Syndrome

This symposium, sponsored by the T21RS's Clinical Committee, described existing biospecimen banks and emerging biomarkers available to support translational research in DS. Additionally, the glutamatergic hypothesis for DS and data from a recently concluded clinical trial of the drug memantine was presented as an example of the many challenges and opportunities involved in designing and performing clinical trials based on animal model data.

Ann-Charlotte (Lotta) Granholm (USA) described the DS Biobank Consortium, DSBC, which aggregates, preserves, and distributes biological samples collected from persons with DS and from individuals without DS (which serve as control specimens). The DSBC is a multisite biobank involving 11 participating universities in the United States and Europe and focuses on collecting and studying tissues from persons with DS of all ages. These biological samples can be blood and other biological fluids, cells, brains and other tissues to support research into the mechanisms underlying health and cognitive issues associated with DS. Such knowledge can inform the design of new treatment approaches to these phenotypes and coexisting conditions.

Michael Rafii (USA) considered ways in which data on biomarkers collected from adults with DS can potentially be integrated into the design of clinical trials involving participants with DS. He described two major biomarker groups, including (1) the NIH-funded ABC-DS initiative and (2) the European Horizon 21 Consortium. The ABC-DS was formed in 2015 and developed into a multicenter, longitudinal study. It targets AD in persons with DS and includes analysis of clinical, cognitive, blood and CSF biomarkers, neuroimaging and neuropathology, and genetic modifiers (Handen et al., 2020). The Horizon21 DS Consortium is formed by 10 European centers and is focused on obtaining essential clinical data on the progression of AD in DS, including in its early stages, as well as to develop a clinical trial-ready network (A. Strydom et al., 2018a, 2018b).

Conclusions: Biobanks play a crucial role in trisomy 21 research. Having access to biological samples from individuals with DS and neurotypical controls allows scientists to study novel mechanisms in the context of DS and may guide new treatment approaches. Biomarker research, including initiatives like ABC-DS and Horizon 21, is advancing clinical trial designs by identifying key clinical, cognitive, and biological markers, particularly for AD in DS.

## Dialogues with Industry and Sponsors to Support Clinical Translation in DS

This session introduced several industry partners working on potential treatments for cognitive impairment and AD in DS. The discussion focused on how to best accommodate the needs of people with DS in clinical trials. T21RS will endeavor to support the industry in optimizing clinical trial design.

Eugene Williams (ProMIS Neurosciences, USA and Canada) co-founded a biotechnology company committed to discovering and developing therapeutic antibodies selective for toxic oligomers associated with the development and progression of neurodegenerative and other misfolded protein diseases. He discussed their plans to develop active and passive immunotherapy to treat and prevent AD in DS.

Pier Vincenzo Piazza (Aelis Farma Inc, France) presented their development of a drug candidate called AEF0217, designed explicitly for treating cognitive deficits. The AEF0217 drug candidate has completed the Phase 1 clinical program and will enter Phase 1/2 study in subjects with DS in Europe.

Patrick J. Kesslak (Alzheon, Inc., USA) presented the clinical profile of ALZ-801, an amyloid oligomer inhibitor in late-stage development, and its potential for the DS population. ALZ-801 is administered orally as a tablet and acts on the same pathway as anti-amyloid antibodies but works upstream to prevent the formation of neurotoxic soluble amyloid oligomers without disrupting the insoluble plaque deposits, avoiding the vascular complications of ARIA-E seen with anti-amyloid antibodies.

Tiffany Chow (IQVIA, USA) presented her work with patients and families living with dementia. Her talk focused on modifying existing resources for AD trials and integrating them into DS trials.

Conclusions: Industry collaborations are important in advancing research and treatment options for AD in DS. Industry partners such as ProMIS Neurosciences, Aelis Farma, Alzheon and IQVIA are developing innovative therapies, including immunotherapies and drug candidates to treat AD in DS. These collaborations aim to address the unique needs of DS patients in clinical trials, with the goal of optimizing trial design and treatment effectiveness. This underscores the necessity of ongoing industry involvement to translate research into therapies for individuals with S.

## Challenges and Opportunities for Caregivers

Caregivers are often primary reporters, advocates, and champions for their loved ones with DS. Often, their insight is crucial to understanding the needs of adults with DS. This symposium discussed the challenges of caregivers through data collected from online surveys and a live discussion with caregivers.

### Caregiver's Report: the Desires, Challenges, and Opportunities for their Loved Ones with DS.

Online surveys are an excellent tool for DS research since we can reach people from all over the planet and get a wide range of perspectives. Three timely and critical areas that have significant implications for individuals with DS and caregivers: COVID-19-related changes, research participation, and independence were discussed.

Eric Rubenstein (USA) presented how the COVID-19 pandemic affected individuals with DS one year into the pandemic, April- June 2021. Adults with DS lost enrichment activities, had increased negative behavioral changes, and struggled with employment. Twenty percent reported less healthcare usage, 20% reported delayed routine care and 86.5% lost activities. As the pandemic continues, targeted support for adults with DS is needed to prevent further skill loss and mental health conditions.

Nichole Kyprianou (USA) presented caregiver perspectives toward research participation for adults with DS. Caregivers reported that the adult with DS they cared for would be more comfortable participating in physiological research, such as research involving fit bits (70.2% would participate), exercise or diet apps. At the same time, they would be less likely to participate in clinical trials involving more invasive procedures such as injections and laboratory exams like MRIs, and there was little difference by age or gender of the adults with DS or caregiver education.

Stephanie Santoro (USA) presented a survey of 408 caregivers of adolescents and adults, describing the current state of factors related to independence, focusing on the importance of independent living skills. The top goals by topic were safety from sexual abuse, communicating wants and needs, toileting independently, living independently / semi-independently, engaging in leisure time appropriately, and reading and writing. Dr Santoro discussed how prioritizing factors for independence can help guide support services and research.

Conclusions: Online surveys on the experiences of caregivers of individuals DS highlighted key challenges and opportunities. The COVID-19 pandemic led to increased behavioral issues, loss of enrichment activities, and delayed healthcare for adults with DS. Caregivers expressed that individuals with DS had greater comfort with physiological research, but less interest in clinical trials involving invasive procedures. The survey also emphasized the importance of independent living skills, prioritizing safety, communication, and personal care as key goals for promoting independence in adolescents and adults with DS.

### Meeting Challenges in Caring for those with DS: A Dialogue Between Families/Advocates and the DS Medical Interest Group (DSMIG)

This session was devoted to hearing the voices of advocates and family members regarding real-world concerns regarding health and health care. The meeting was attended by about 50 advocates and caregivers who engendered a robust exchange of ideas. A key point was that attendees were eager to share their ideas with the Society and DSMIG. While there has been a history of engagement with DSMIG, relatively few direct discussions with the society have been held. Nevertheless, the Science and Society sessions have addressed topics of great interest to families, which is quite positive. The most salient takeaway from the meeting was the desire to allow those with DS and their families to access healthcare providers with expertise in caring for people with the syndrome. This is especially the case for adults with DS. Indeed, a recent survey found that less than 5% of all adults with DS were provided care by those knowledgeable about the increased frequency of conditions in DS. The result is that families find their caregivers have little expertise in the diagnosis, treatment or significance of the medical issues that confront those with this condition.

Conclusions: The strong sense emerging from the meeting was that society can play a helpful and supportive role in continuing to create a dialogue between families and healthcare professionals. Moreover, an effort to increase access to expert care would be greatly appreciated. The DSMIG will continue to address the concerns of advocates and families and hopes to have a session at each of our meetings to hear their voices.

## Awards

### Montserrat Trueta Outstanding Career Award

This award is supported by the Catalan DS Foundation (FCSD). Montserrat Trueta founded the FCSD, an organization born out of a common interest shared by parents and professionals alike, to gain a better understanding of DS. FCSD supports scientific research and applied work in the fields of education, psychology, medicine, and welfare. From its beginning, outstanding international scientists specializing in DS research provided the FCSD with their valuable advice. This award recognizes outstanding scientists in the field of DS for their sustained and distinguished careers, including groundbreaking scientific contributions, leadership, and mentoring. The award reflects the strong alliance that FCDS is building with the T21RS community.

Ira Lott was presented with the Montserrat Trueta Outstanding Career Award. Lott has a strong history of advocacy and care for people with DS and was involved early in the medical aspects of the Special Olympics when he was clinical director of the Eunice Kennedy Shriver Center.

### Dissertation Awards

T21RS formally honored the exemplary merit and exceptional quality of the research of two recently graduated doctoral students by awarding the Annette Karmiloff-Smith and Michael Harpold Thesis Awards. The winners of the "Annette Karmiloff-Smith Thesis Award Program and Michael Harpold Dissertation Award" for outstanding Ph.D. thesis launched in 2021 were Anna Joyce Moyer and Tomer Illouz. Moyer, from the University of Alabama in Birmingham, presented her PhD thesis entitled "Overexpression screen of Hsa21 genes reveals modulators of Sonic hedgehog signaling relevant to DS". Illouz, from the Eitan Okun lab, Bar-ILan University, Ramat-Gan, Israel, presented his work "Targeting Amyloid-β in mouse models of DS and Alzheimer's disease: A role for microglial and glial cells."

### Trainees "Blitz" Elevator Pitch and Poster Session

A special blitz presentation session for junior investigators was organized. From all the posters, the program committee selected twenty among those submitted by young investigators to be presented in the blitz session, according to scientific interest and geographical and gender diversity. The winners of the poster awards were Daniella Balduino Victorino (ICM, Paris, France) for her work "Preclinical comparison of the pharmacokinetics and efficacy of two selective negative allosteric modulators of GABAARs containing alpha5 subunits on synaptic inhibition and behavioral function" and Agnish Ganguly (University of Calcutta, India) for his work "Association of RFC1 A80G Polymorphism with the incidence of congenital heart defects in individuals with DS from Indian Bengali-speaking population".

## Science and Society

The T21RS committee for Science and Society aims to approach science to people and promote access to research for individuals with DS. At each T21RS International Conference, there is a Science and Society Symposium dedicated to individuals with DS and their families, providing a space for interaction and communication between them and the attending scientists. To encourage the participation of people with DS and their families, this edition's symposium was in hybrid form, with presentations in English translated simultaneously into French and Spanish by professional interpreters.

Over 180 people virtually attended the symposium simultaneously with the onsite conference attendees. The symposium was also recorded and made available online at the T21RS website. We recorded two short videos summarizing the highlights of the conference and testimonials from research participants. The symposium was partly facilitated by people with DS: Theresa Mabie (opening), a panel of actors with DS from Hollywood (Cole Sibus, Blair Williamson, Gail Williamson), a panel of persons with DS and their families about research participation (testimonials), Sujeet Desai (music show and closing). The symposium also included a panel discussion on research participation, involving 7 experts as speakers, and 4 scientific presentations aimed at the general audience. The topics discussed were chosen based on their interest in improving the health of people with DS: COVID-19, regressive syndrome, physical activity and sleep apnea disease.

Sujay Ghosh (India) highlighted that the presentation and outcomes of COVID-19 among individuals with DS are more severe for patients from India than for those from high-income countries and that global efforts should especially target vaccination campaigns and other risk-reducing interventions for individuals with DS from low-income countries.

Jonathan Santoro (USA) described that intravenous immunoglobulin could be highly effective in treating DS regression disorder and that individuals with a history of personal autoimmunity or neurodiagnostic abnormalities were more likely to relapse following weaning of immunotherapy, indicating the potential for a chronic autoimmune etiology in some cases of this disorder.

In the second slot for scientific presentations, Sarah Pape (UK) focused her talk on the fact that regular moderate and high-intensity exercise could reduce the risk of clinically detectable decline in the DS population and how important it is that people with DS are supported to be physically active and to promote exercise as part of a healthy aging plan.

Starting with Angela Britton—on behalf of Hampus Hillerstrom (LuMind IDSC, USA)-talked about the importance and need for research participation of people with DS, highlighting the disparities that this population suffers and the frequent willingness of families to participate in research.

Several Clinical Trial Networks were introduced in this session. Angela Britton (LuMind IDSC, USA) presented the DS Clinical Trials Network (DS-CTN), which was built to conduct large-scale, multisite trials under the supervision of experienced clinicians who are experts at providing medical care for individuals with DS in the US. Michael Rafii (USA) presented the ACTC-DS & Trial Ready Cohort (TRC-DS), a setting for matching people with DS to clinical trials related to AD in the USA and Europe. Andre Strydom (UK) introduced the Horizon 21 consortium, a European research network aiming to harmonize the cognitive assessment of adults with DS, bringing new biomarkers into the clinic, understanding variability in risk for decline, and establishing a clinical trials network.

Afterwards, Lotta Granholm (USA) explained the importance of a brain bank, the motivations of brain donation and how to participate. Marie-Claude Potier (France) and Mara Dierssen (Spain) presented the Trisomy 21 Cluster, a two-year networking action to enhance the coordination of DS research in Europe funded by EBRA, the European Brain Research Area.

Finally, a video was displayed expressing the testimonials of several adults with DS who had participated in research and their families, coordinated by Isabel Barroeta (Spain). To conclude, all the experts in this panel had a discussion, getting through the topics proposed by the audience.

## Conclusion

Ultimately, sharing information in the T21RS meeting is an inspiration for new and junior investigators to join our effort to a future where we can implement preventions and treatments for various health and societal challenges that affect people with DS. Forging links between those who engage in research and care with those they care for will inform societal efforts and enable them to be even more impactful. The meeting provided opportunities to engage in this important dialogue and to highlight the amazing accomplishments of people with DS. Toward this goal, in addition to the Science and Society session, a virtual session was held during the meeting at which time we fostered and developed this dialogue.

## Data Availability

No datasets were generated or analysed during the current study.

## Abbreviations

ABC-DS: Alzheimer Biomarker Consortium-DS
ACTC-DS: Alzheimer's Clinical Trials Consortium-Down syndrome
AD: Alzheimer’s disease
ADHD: Attention deficit hyperactivity disorder
Akt: Ak strain transforming
ALL: Acute lymphoblastic leukemia
AML: Acute myeloid leukemia
AMPK: AMP-activated protein kinase
APP: Amyloid precursor protein
ARRIVE: Animal research: reporting of in vivo experiments
ASD: Autism spectrum disorder
Aβ: Amyloid-beta
APP: Amyloid precursor protein
BMI: Body mass index
CA1: Cornu ammonis 1
CAA: Cerebral amyloid angiopathy CAA
CAR: Chimeric antigen receptor
CDD: CDKL5 deficiency disorder
CDKL5: Cyclin-dependent kinase-like 5
CN-PiB: Cyano-PiB
COVID-19: Coronavirus disease 2019
CRLF2-R: Cytokine receptor-like factor 2 rearrangements
CRT: Cued recall test
CSF: Cerebrospinal fluid
CTF: C-terminal fragment
D21: Disomic
DNA: Deoxyribonucleic acid
DS: Down syndrome
DSAD: Down syndrome with Alzheimer’s disease
DSBC: Down syndrome Biobank Consortium
DS-CTN: Down syndrome clinical trials network
DSMIG: DS Medical Interest Group
DSMSE: Down syndrome mental status exam
DUP-APP: Familial cases with *APP* duplication
Dyrk1A: Dual specificity tyrosine phosphorylation regulated kinase 1A
EBRA: European brain research area
ELS: Expressive language sampling
EMBO: European Molecular Biology Organisation
EV: Extracellular vesicles
FCSD: Catalan Down syndrome Foundation
FDA: Food and Drug Administration
FXS: Fragile X
GABA: Gamma-aminobutyric acid
GABAAR: Gamma-aminobutyric acid receptor
GAM: Genome architecture mapping
GATA1: GATA binding factor 1
GO-DS21: Gene Overdosage and comorbidities during the early lifetime in Down syndrome
Hsa21: Human chromosome 21
IFN: Interferon
IFNARs: Encoded type I interferon receptor genes
IFNR: Interferon receptor
IGF1: Insulin growth factor 1
iPSC: Induced pluripotent stem cells
iPSC-d-ECs: Endothelial cells derived from iPSC lines
IRS: Insulin receptor substrate
JAK/STAT: Janus kinase/signal transducers and activators of transcription
LC-MS: Liquid chromatography-mass spectrometry
MALDI: Matrix-assisted laser desorption/ionization
ML-DS: Myeloid leukemia of Down syndrome
MRI: Magnetic resonance imaging
mRNA: Messenger ribonucleic acid
mTOR: Mammalian target of rapamycin
NDDs: Neurodevelopmental disorders
NDEVs: Neuron-derived extracellular vesicles
NIH: National Institutes of Health
NRIP1: Nuclear receptor-interacting protein 1
OSA: Obstructive sleep apnea
PAP: Positive airway pressure
PET: Positron emission tomography
PGC-1α: Peroxisome proliferator-activated receptor-gamma coactivator-1alpha
PiB: Pittsburgh Compound B
p-tau: Phosphorylated tau
PTEN: Phosphatase and tensin homolog deleted on chromosome 10
Rab5: Ras-related protein 5
RNA: Ribonucleic acid
SAH: *S*-adenosyl-homocysteine
SAM: *S*-adenosyl methionine
SARS-cov2: Severe acute respiratory syndrome coronavirus 2
SIL: Stable isotope labeled SIL
SIRT1: Sirtuin 1
snRNA-seq: Single nucleus RNA sequencing
T21: Trisomy 21
T21RS: Trisomy 21 Research Society
TAM: Transient abnormal myelopoiesis
THF: Tetrahydrofolate
TNFα: Tumor necrosis factor-alpha
TRC-DS: Trial ready cohort Down syndrome
